# The Role of Astrocytes in Metabolism and Neurotoxicity of the Pyrrolizidine Alkaloid Monocrotaline, the Main Toxin of *Crotalaria retusa*

**DOI:** 10.3389/fphar.2012.00144

**Published:** 2012-08-03

**Authors:** Bruno Penas Seara Pitanga, Ravena P. Nascimento, Victor Diógenes A. Silva, Silvia L. Costa

**Affiliations:** ^1^Laboratório de Neuroquímica e Biologia Celular, Instituto de Ciências da Saúde, Universidade Federal da BahiaSalvador, Brazil

**Keywords:** *Crotalaria*, pyrrolizidine alkaloid, monocrotaline, neurotoxicity, astrocyte, neuron, P450, GSH

## Abstract

The metabolic interactions and signaling between neurons and glial cells are necessary for the development and maintenance of brain functions and structures and for neuroprotection, which includes protection from chemical attack. Astrocytes are essential for cerebral detoxification and present an efficient and specific cytochrome P450 enzymatic system. Whilst *Crotalaria* (Fabaceae, Leguminosae) plants are used in popular medicine, they are considered toxic and can cause damage to livestock and human health problems. Studies in animals have shown cases of poisoning by plants from the genus *Crotalaria*, which induced damage to the central nervous system. This finding has been attributed to the toxic effects of the pyrrolizidine alkaloid (PA) monocrotaline (MCT). The involvement of P450 enzymatic systems in MCT hepatic and pulmonary metabolism and toxicity has been elucidated, but little is known about the pathways implicated in the bioactivation of these systems and the direct contribution of these systems to brain toxicity. This review will present the main toxicological aspects of the *Crotalaria* genus that are established in the literature and recent findings describing the mechanisms involved in the neurotoxic effects of MCT, which was extracted from *Crotalaria retusa*, and its interaction with neurons in isolated astrocytes.

## Neurotoxic Plants of Veterinary and Human Interest

In natural conditions, poisonous plants are introduced into the body of man or domestic animals and are capable of causing damage, which is reflected in the health and vitality of these beings. They cause an imbalance, which results in symptoms of intoxication in the patient. The toxic agent of a plant consists of a substance or a set of chemically well-defined substances, and the latter are of the same nature or different. When these substances contact the body, they are capable of causing intoxication. When poisonous plants are eaten by herbivores, many of them share space with grass, or they can be eaten accidentally by humans; the consumption of poisonous plants can result in nerve disturbances in the human digestive tract and heart and in some cases, lead to death. In Brazil, because data on the frequency and causes of mortality from some states are lacking, it is difficult to estimate losses due to the death of animals. According to Riet-Correa and Medeiros (2001), in the states of Rio Grande do Sul and Santa Catarina, the annual mortality rate of cattle caused by toxic plants is 10–14%. With regard to arid regions of the country, the situation is more severe during periods when food is scarce because animals tend to eat what is available. In those regions, the annual mortality rate is 7.2%. In Brazil, the resulting impact on animal production amounts to millions of dollars. Another important aspect related to the ingestion of toxic plants by animals is that the toxins can be transferred to humans through the consumption of milk, meat, eggs, or other animal products. It is well known that in the United States, the consumption of milk produced by cows that are maintained in pastures invaded by *Eupatorium rugosum* causes a disease condition known as “milk sickness,” which may result in human death (Panter and James, 1990). Other toxins in milk include pyrrolizidine alkaloids, which are present in plants from the genera *Senecio*, *Heliotropium*, *Echium*, *Amsinckia*, *Symphytum* (comfrey), *Cynoglossum* (hound’s tongue), and *Festuca* (tall fescue) and are abundant in plants from the genus *Crotalaria* (Dickinson et al., 1976; Panter and James, 1990). In Brazil, the milk from goats fed with *Crotalaria spectabilis* was toxic to rats (Medeiros et al., 1999), and litters of rats fed with *C. spectabilis* or monocrotaline (MCT), the main pyrrolizidine alkaloid, had poisoned milk (Medeiros et al., 1998). Plants that have been considered neurotoxic are described in Table 1 and include the genera *Ipomoea, Ricinus*, *Phalaris*, *Solanum*, *Prosopis*, and *Crotalaria*.

**Table 1:** **Plants that have been considered as neurotoxic and toxic metabolites**.

Plant species	Neurotoxic metabolite	Reference
Crotalaria	Monocrotaline and tricodesmine (alkaloids)	Riet-Correa et al. (2011)
*C. spectabilis*	
*C. crispate*	
*C. dura*	
*C. mucronata*	
*C. retusa*	
*Ipomoea asarifolia*	Lectins not yet characterized	Salles et al. (2011)
Phalaris sp. (Poaceae)	Tryptamine (alkaloid)	Cantón et al. (2010)
*P. angusta*	
*P. angusta*	
*P. aquatica*	
*P. arundinacea*	
*P. brachystachys*	
*P. canariensis*	
*P. paradoxa*	
*P. caroliniana*	
*P. minor*	
*Prosopis juliflora*	Juliprosine and juliprosopine (alkaloids)	Choudhary et al. (2005)
*Ricinus communis* (leaves)	Ricinine (alkaloid)	Worbs et al. (2011)
*Solanum fastigiatum*	Alkaloids not yet characterized	Rech et al. (2006)

## Astrocytes: An Efficient System for Detoxification and Bioactivation in the CNS

The metabolic interactions and signaling between neurons and glial cells have been previously described. These intimate interactions are necessary for the development and maintenance of brain functions and structures and for neuroprotection, which includes protection induced by chemical attack (Tardy, 2002; Eskes et al., 2003; Zurich et al., 2004). The interrelationships between neural and glial cells contribute to the development, function, and reparative capacity of the brain and can participate in its deterioration due to aging or disease (for review see Tardy, 2002, and Sofroniew and Vinters, 2010). In the Central Nervous System (CNS), astrocytes, and microglia are the two cell populations capable of responding to neuronal injuries. These cells can change morphology, alter the expression patterns of neurotrophic and/or neurotoxic factors, or affect the association between these two phenomena (Streit et al., 1999). Astrocytes and microglia respond to all forms of neurological damage, including those induced by toxicants undergoing activation, a phenomenon known as gliosis. Astrogliosis is associated with an altered phenotype due to up-regulation of a large number of molecules (Eddleston and Mucke, 1993; Cookson and Pentreath, 1994; Lefrançois et al., 1997; Mead and Pentreath, 1998; Costa et al., 2002; Tardy, 2002), including the accumulation of intermediate filaments containing glial fibrillary acidic protein (GFAP). Several studies have shown that GFAP is up-regulated after exposure to a diverse set of toxic chemicals that includes kainic acid, mercury chloride, aluminum chloride, toluene, ethanol, dibutyryl-cAMP, piperidine alkaloids, and trimethyltin (Rataboul et al., 1989; Cookson and Pentreath, 1994; Mead and Pentreath, 1998; Harry et al., 2002; Hughes et al., 2006; Silva et al., 2007). Although an increase in GFAP expression can be associated with astrogliosis, reactions to physical damages, and even neurodegeneration (Tardy, 1991; Coyle and Schwarcz, 2000; Costa et al., 2002), a reduction in GFAP expression can reflect abnormal synaptogenesis and neurotransmission (O’Callaghan and Jensen, 1992; Moises et al., 2002; Rajkowska et al., 2002).

Many toxic chemicals have undesirable characteristics because of highly reactive metabolic products that are generated within a target organ; this process is known as bioactivation. On rare occasions, chemical bioactivation occurs within the liver, and the toxic products are subsequently transported to the target organ via circulation. In some species, brain tissue contains chemical-activating enzymatic systems, which are unique to (or more active in) this organ when compared with other tissues.

Cytochrome P450, which was named by Omura and Sato (1962), has an important role in the detoxification of xenobiotics for subsequent body elimination (Dutheil et al., 2007). In this process, after contact between a foreign substance and the body, two pathways are possible: the substance is removed, or it is biotransformed into an active compound that is capable of damaging the body (Orellana and Guajardo, 2004). A variety of tissues have the P450 system: liver, kidneys, lungs, skin, intestines, adrenal cortex, testes, placenta, and brain, which is where the bioactivation of compounds that cross the blood brain barrier (BBB) is especially important (Orellana and Guajardo, 2004; Dutheil et al., 2007).

In addition to providing nutritional and structural support to neurons in the CNS, one of the most important functions of astrocytes is to control the neurotoxins inside of the CNS. The brain is the target of numerous toxic compounds, such as lipophilic organic solvents and psychoactive drugs, which include amphetamines, benzodiazepines, and alkaloids, such as cocaine. These substances can cross the BBB to exert their action at the central level. The *in situ* metabolism of these substances leads to local pharmacological modulation and the transformation of psychotropic drugs into hydrophilic compounds by cytochrome P450 in the brain, which results in slower elimination (Ravindranath, 1998). A variety of enzymatic systems also have this capacity, including the active CYP450 system (Coyle and Schwarcz, 2000; Tardy, 2002). Astrocytes are the first line of defense against xenobiotics, and they express high levels of P450, which are approximately two point seven times greater than the levels found in neurons, which indicates that neuronal P450 does not function the same as astrocytic P450. Studies of the brain’s bioactivation systems have revealed that astrocyte-specific cytochrome P450-dependent monooxygenases play a causative role in establishing the brain as the target organ of several toxic agents (Meyer et al., 2001). The P450 isoforms CYP1A1, CYP1A1/2, CYP2B1, CYP2B6, CYP2C11, CYP2C, CYP2D6, CYP2E1, and CYP3A were determined in rat and human astrocytes and considered functionally active (for review, see Malaplate-Armand et al., 2004; Meyer et al., 2007).

## Toxicological Aspects of *Crotalaria*

Plants of the *Crotalaria* genus grow abundantly in tropical and subtropical zones and are adopted in popular medicine (Atal and Sawhney, 1973; Mattocks, 1986). Because *Crotalaria* are invasive plants, they are commonly found in grain plantations and pastures (Cheeke, 1988), and they can be accidentally ingested by humans and animals. In Brazil, approximately 40 species have been found and are often eaten by animals, especially during food shortages (Tokarnia et al. (2000)). These plants are rich in pyrrolizidine alkaloids (PAs), which are the main toxins derived from plants that are transferred to humans and animals (Mattocks, 1986; Huxtable, 1990). In animals, intoxications from *Crotalaria* have been described in many countries (Boghossian et al., 2007), including the Brazilian states of Mato Grosso do Sul (Lemos and Barros, 1998), Minas Gerais (Nobre et al., 1994), and Paraiba (Nobre et al., 2004a,b, 2005). In the literature, there are descriptions of intoxications in equines (Gibbons et al., 1953; Gardiner et al., 1965), bovines (Barri and Adam, 1981), pigs (Peckham et al., 1974; Souza et al., 1997), birds (Norton and O’Rourke, 1979), and caprines (Barri et al., 1984). However, bovines and equines are more susceptible to PA intoxication because they are 30–40 times more susceptible to PA than ovines and caprines (Riet-Correa et al., 2006). In Australia, *Crotalaria retusa* and *C. crispata* were responsible for the disease known as “Kimberly horse disease” or “walkabout disease” (Rose et al., 1957). In the semi-arid region of Paraíba State in northeastern Brazil, cases of acute intoxication in horses from *C. retusa* seeds occurred during the dry season (Nobre et al., 2004a). In humans, intoxication from *Crotalaria* can occur because of the consumption of grains contaminated by plant seeds (Huxtable, 1989) and its use in popular medicine (Atal and Sawhney, 1973). In India, for example, this plant is used for the treatment of scabies and impetigo (a contagious skin disease caused by *Staphylococcus* and *Streptococcus*; Damron and Jacob, 2001). Moreover, animals that feed on large quantities of *Crotalaria* can eliminate the secondary metabolite through milk, which is potentially dangerous for nurslings and individuals who ingest contaminated milk (Panter and James, 1990).

However, it is well known that to exert their toxic effect, PAs need to be metabolized by the hepatic and lung enzymatic P450 cytochrome system, which generates active metabolites, such as dehydroalkaloids and pyrrols (Mattocks, 1986; Couet et al., 1996; Kasahara et al., 1997). These metabolites might affect cellular macromolecules, such as DNA and proteins, and form adducts that can initiate an acute or chronic toxicity (Culvenor et al., 1962). Monocrotaline is the major PA of *C. retusa* and is responsible for the damaging effects observed in different animal species (Cheecke, 1998). This alkali is primarily hepatotoxic and pneumotoxic, but nefrotoxic, cardiotoxic, fetotoxic, neurotoxic, and carcinogenic effects are also related to MCT intoxication (Mattocks, 1986; Ribeiro et al., 1993; Thomas et al., 1996; Cheecke, 1998; Medeiros et al., 2000; Lin et al., 2001; Wang et al., 2005). In fact, tissue-bound pyrrolic metabolites were detected in the liver, lung, heart, and kidney after intraperitoneal injection of MCT (Yan and Huxtable, 1995). Animal species that are particularly vulnerable, such as horses, show classic liver fibrosis and neurological symptoms (Kimberly Horse Disease) that are associated with chronic MCT exposure (Rose et al., 1957; Nobre et al., 2004a).

Studies of MCT metabolism in the liver and lungs of rats intoxicated with MCT show that after the alkaloids are metabolized by the P450 cytochrome system, they undergo dehydrogenation to produce dehydromonocrotaline (DHMC), which is considered a highly toxic compound (Lin et al., 2001; Wang et al., 2005). In humans, both forms of detoxification, namely activation and reaction, are catalyzed by the CYP 3A4 cytochrome (Miranda et al., 1991). However, the resulting DHMC is unstable and can continue through several metabolic pathways: (1) hydrolysis to 6,7-dihydro-7-hydroxy-1-hydroxymethyl-5H-pyirrolizine (DHP), one of the major active metabolites; (2) conjugation with glutathione (GSH) in the liver to form 7-enamtiomers glutationil-6,7-dihydro-1-hydroxymethyl-5H-pirrolizine (7-GS-DHP) and 7,9-DP-diGSH; (3) nucleophilic alkylation of cellular macromolecules, which is a process that highlights the toxic activity of DHMC; (4) or release by circulation (Wang et al., 2005). MCT can also be biotransformed by the P450 system to generate N-oxides, which can be hydrolyzed into DHP, a major reactive metabolite, or converted into a dehydroalkaloid. In the model of MCT-induced progressive pulmonary hypertension (MCT-PH), some molecular targets and pathways have been identified. In MCT-PH, the protein levels of angiotensin-1 (Ang1), Ang2, endothelial nitric oxide synthase (eNOS), inducible NOS (iNOS), heme oxygenase 1 (HO1), and vascular endothelial growth factor (VEGF) were increased (Cho et al., 2009). MCT-PH was also associated with increased expression of the matrix metalloproteinases (MMP)-2 and MMP-9 and the protein psmad2, which is involved in transforming growth factor-β (TGF-β) signaling (Zaiman et al., 2008). The protein expression and activity of pulmonary soluble epoxide hydrolase (sEH), which is involved in the metabolism of epoxyeicosatrienoic acids (EETs), and the activity of pulmonary cytochrome P450 epoxygenase were impaired during MCT-PH in rats (Revermann et al., 2009).

The presence of clinical neurological signs in animals intoxicated with *Crotalaria* was initially associated with hepatic encephalopathy because urea metabolism followed by hyperammonemia was impossible (Cheeke, 1988; Nobre et al., 2004b). Moreover, the metabolites derived from the alkaloids trichodesmine and MCT, dehydrotrichodesmine and DHMC, respectively, were found and measured in the brains of rats that were experimentally intoxicated. These results demonstrated the ability of these molecules to cross the BBB (Yan and Huxtable, 1995) and suggest that the neurological signs observed in intoxicated animals result from efficient metabolism of the PA from *Crotalaria* into active components of CNS cells. The hepatic P450 enzymatic systems that are involved in MCT metabolism related to hepatic and pulmonary toxicity have been elucidated; however, little is known about the pathways acting in the bioactivation of these systems or about their direct contribution to brain toxicity.

## Understanding the MCT Metabolism of Astrocytes and Its Relationship with Neurotoxicity

Primary cultures of astrocytes and neurons and co-cultures of neuronal/glial cells, which are derived from the cortex of neonatal or embryonic rats or mice, are reliable *in vitro* models for the biological and biochemical study of CNS cells in normal or pathological conditions. These models have been used for decades and have also been adopted as models for the study of neurotoxic substances (Booher and Sensenbrenner, 1972; Lesuisse and Martin, 2001). To clarify the neurotoxic effects of MCT that was extracted from *C. retusa* and its DHMC derivative, we conducted *in vitro* studies in primary cultures of astrocytes or neurons and in primary co-cultures of astrocytes/neurons obtained from the cerebral cortex of Wistar rats.

Using an MTT test that measures mitochondrial function, we noticed that MCT concentrations ranging from 0.1 to 500 μM did not induce cytotoxicity in these cells (Barreto et al., 2006). In contrast, 1 μM DHMC reduced cell viability 24 h after treatment, and this reduction was more significant after 72 h of treatment (Barreto et al., 2008). Metabolites, such as dehydroalkaloids and pyrrols, might alkylate cellular macromolecules, such as proteins and DNA, to form adducts that can initiate an acute or chronic toxicity (Culvenor et al., 1962). We also observed that both MCT and DHMC induced changes in the expression levels and patterns of GFAP, a major protein of the cytoskeleton in astrocytes. This phenomenon was accompanied by severe phenotypic changes and by hypertrophic astrocytes that characterized the cells reactive to these alkaloids (Barreto et al., 2008). Moreover, in another study, we investigated the action of MCT in glial cells of the GL-15 strain and demonstrated that MCT and DHMC interfered with cellular growth and induction of megalocytosis, induced significant down-regulation of proteins associated with microtubules (MAPs), and presented a genotoxic property (Silva-Neto et al., 2010).

The interactions between neurons and glial cells, such as astrocytes, are essential for synaptic properties (Volterra and Meldolesi, 2005), development, homeostasis, and detoxification of the CNS (Letournel-Boulland et al., 1994); in contrast, neurons contribute to the proliferation and survival of glial cells (Gomes et al., 2001). Hence, we aimed to clarify the complexity of neurotoxic phenomena induced by MCT in different populations of CNS cells; thus, we recently conducted another study in co-cultures of neurons/astrocytes and in primary cultures of isolated neurons. Using MTT testing, we observed a reduction in cell viability after 72 h of treatment with 100 μM MCT. Under these conditions, the toxicity of MCT was also demonstrated by determining the levels of lactate dehydrogenase activity in the supernatants of co-cultures, which indicated plasma membrane damage had occurred. However, the changes in cell viability were not observed in primary cultures of isolated neurons. One of the main features of astrogliosis is an increase in GFAP expression (Tardy, 2002). In our system of co-cultures, when both astrocytes and neurons were in contact, vacuolization, and an increase in the cell body of astrocytes were observed; an increase in GFAP expression after 72 h treatment with 100 μM MCT was also observed and indicated astrogliosis. The resistance of astrocytes to MCT toxicity in astrocytes isolated from primary cultures, observed by Barreto et al. (2006), can be understood as the result of their limited capacity in metabolizing xenobiotics, which must be restored when neurons and glia interact. Mingatto et al. (2007) worked with isolated mitochondria from rat liver and observed a decrease in the NADH oxidase activity of the respiratory chain complex I. Thus, MCT interference in mitochondrial function and toxicity might result from the generation of active metabolites, such as DHMC, by astrocytes when these cells interact with neurons. Moreover, it is known that when large quantities of certain toxic compounds circulate, *detoxifying* enzymes, such as cytochrome P450 (from the smooth cytoplasmic reticulum), are produced, which causes folding of the organellar surface area (Alberts et al., 2002). Once the toxin disappears, the excess smooth endoplasmic reticulum membrane is removed by a process dependent on lysosomes, called autophagocytosis. Cytoplasmic vacuoles were evident in co-cultures of astrocytes/neurons (Pitanga et al., 2011) but not in primary cultures of astrocytes treated with MCT (Barreto et al., 2006), which confirms the hypothesis that metabolic properties of MCT are dependent on interactions between neurons and glia.

In association with MAPs, neuronal β-tubulin III protein, which is considered the main structural protein of neurons (Lesuisse and Martin, 2001), integrates a dynamic network and plays a crucial role in key biological processes, such as cell division and intracellular transport (Guzik and Goldstein, 2004). By applying immunocytochemistry in co-cultures exposed to 10–100 μM MCT, we found that MCT disrupts β-tubulin III staining, which suggested that the polymerization of this protein failed in neurons. Moreover, by western blot analysis, we observed a strong decrease in the β-tubulin III expression in protein extracts, which indicated protein destabilization had occurred (Pitanga et al., 2011). These findings support the theory that the neuronal β-tubulin III cytoskeletal protein is a molecular target of MCT or its metabolites, which might interfere with the dynamics of stabilization of microtubular proteins.

As previously discussed, the cytotoxicity of MCT in hepatocytes is due to the generation of DHMC by the cytochrome P450 enzyme complex; the resulting DHMC, then binds to GSH and causes its depletion (Lin et al., 2002; Wang et al., 2005). Studies indicate that GSH protects glial cells from oxidative stress, and GSH depletion causes several biochemical and molecular changes that might be related to neurological disorders (Lee et al., 2010). We also observed that treatment with 100 μM MCT depleted GSH in co-cultures of astrocytes/neurons and that the GSH depletion was reversed when the cytochrome P450 enzymatic system was inhibited by cimetidine, a potent inhibitor of hepatic cytochrome P450 (Chang et al. (1992)). These findings indicate that GSH depletion is dependent on the metabolism and synthesis of active metabolites via the P450 system and that this phenomenon is involved in MCT-induced neurotoxicity (Pitanga et al., 2011). Taken together, these *in vitro* studies of primary rat cultures of astrocytes or neurons provide new information on the mechanisms of MCT toxicity in different cell populations in the CNS and their relationship to neurological phenomena observed in intoxicated animals.

**Figure 1:**
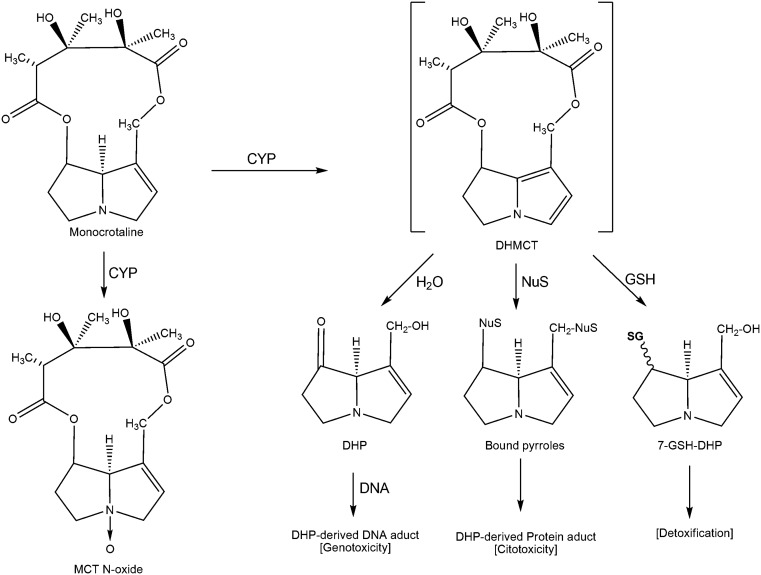
**The proposed metabolic activation and detoxification pathways of monocrotaline in astrocytes interacting with neurons**. CYP, enzyme of the cytochrome P450 complex; GSH, glutathione; DHMC, dehydromonocrotaline; DHP, 6,7-dihydro-7-hydroxy-1-hydroxymethyl-5H-pyrrolizine; 7-GSH-DHP, 7-glutationil-6, 7-dihydro-1-hydroxymethyl-5H-pyrrolizine; 7.9-7.9-DHP-diGSH diglutationil-6,7-dihydro-1-hydroxymethyl-5H-pyrrolizine; NuS, nucleophilic biological macromolecules.

To induce hepatotoxicity and pneumotoxicity, PAs, such as MCT, must be metabolized into active metabolites by the P450 system of the liver or lungs (Lin et al., 2001; Wang et al., 2005). Because of this requirement and our findings, we propose a metabolic mechanism for MCT in isolated astrocytes and in interaction with neurons. We propose that MCT acts through an efficient P450 system to generate greater amounts of active metabolites, including DHMC, monocrotaline-N-oxide, and derivatives, such as DHP; these active metabolites then induce cellular toxicity or are detoxified by their conjugation with GSH (Figure 1). The pyrrolic metabolite DHMC is a highly unstable, intermediate reactive metabolite and that can: (i) undergo hydrolysis to form DHP and subsequently form the DHP-derived DNA adducts by a reaction with DNA; (ii) bind to cellular DNA and subsequently be hydrolyzed to form the DHP-derived DNA adducts; or (iii) conjugate with nucleophilic biological macromolecules, such as cellular proteins, and with GSH. We predict that the reversion of cellular toxicity in the primary cultures of astrocytes/neurons after exposure to the potent CYP inhibitor cimetidine indicates that P450 metabolism and DHMC, DHP, and/or N-oxide formation are essential for MCT toxicity in CNS cells. Finally, because MCT and DHMC target specific neural cytoskeletal proteins, such as GFAP and β-tubulin III, and because of our biological profiles of PAs, mainly MCT and their derivatives, these natural products should be considered potential lead compounds for drug development to treat CNS disorders, such as cancer.

## Conflict of Interest Statement

The authors declare that the research was conducted in the absence of any commercial or financial relationships that could be construed as a potential conflict of interest.
